# The ecology of plasmid-coded antibiotic resistance: a basic framework for experimental research and modeling

**DOI:** 10.1016/j.csbj.2020.12.027

**Published:** 2020-12-29

**Authors:** Martin Zwanzig

**Affiliations:** Faculty of Environmental Sciences, Technische Universität Dresden, Pienner Str. 8, D-01737 Tharandt, Germany

**Keywords:** Plasmid ecology, Antibiotic resistance, Horizontal gene transfer, Fitness, Agent-based modeling, Simulation model, Individual heterogeneity, Evolution, Biotic interactions

## Abstract

Many antibiotic resistance genes are associated with plasmids. The ecological success of these mobile genetic elements within microbial communities depends on varying mechanisms to secure their own propagation, not only on environmental selection. Among the most important are the cost of plasmids and their ability to be transferred to new hosts through mechanisms such as conjugation. These are regulated by dynamic control systems of the conjugation machinery and genetic adaptations that plasmid-host pairs can acquire in coevolution. However, in complex communities, these processes and mechanisms are subject to a variety of interactions with other bacterial species and other plasmid types. This article summarizes basic plasmid properties and ecological principles particularly important for understanding the persistence of plasmid-coded antibiotic resistance in aquatic environments. Through selected examples, it further introduces to the features of different types of simulation models such as systems of ordinary differential equations and individual-based models, which are considered to be important tools to understand these complex systems. This ecological perspective aims to improve the way we study and understand the dynamics, diversity and persistence of plasmids and associated antibiotic resistance genes.

## Highlights

• Understanding plasmid-coded antibiotic resistance from an ecological perspective.
• Plasmid fitness considering complex biotic interactions and evolutionary modifications.
• Comparison of biofilm versus plankton and artificial versus natural environments.
• Introduction and brief review of population-level and individual-based models.

## Graphical abstract

**Figure:**
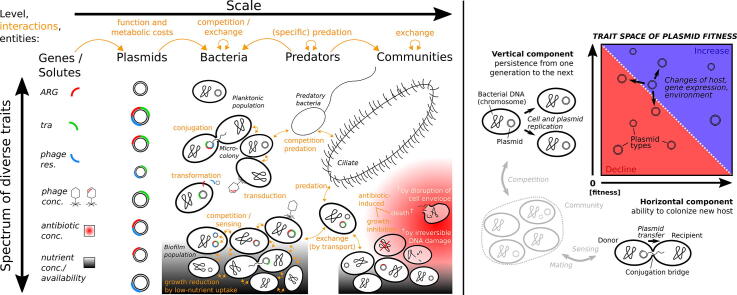

## 1 Introduction

Antibiotic resistances are spreading worldwide in microbial communities and environments [1]. They are a threat to human health, since the use of antibiotics is often the only way to treat infectious diseases [2].

Some bacteria are resistant to antibiotics due to intrinsic structural or functional characteristics [3]. Other are able to develop or acquire resistance mechanisms through mutation or horizontal gene transfer (HGT) [4]. The latter refers, for example, to the spread of mobile genetic elements (MGE) such as plasmids by cell-to-cell contact of bacteria. Other MGE and potential sources of antibotic resistance are bacteriophages, genomic islands, insertion sequence elements, transposons and integrons [5]. Among MGE, conjugative plasmids are the most significant for HGT [6], [7] and considered to be major drivers for the dissemination of antibiotic resistance genes among clinically relevant pathogens [8]. Studies comparing collections of bacterial pathogens before and after the era of antibiotic use showed that plasmids were similarly common, but resistance genes were not, suggesting that they developed in bacteria and were inserted into existing plasmids [9].

*De novo* resistances to antibiotics develop by both DNA mutations and adaptation of expression levels [10]. This includes for example efflux-pump activations enabling the survival at low levels of antibiotics. Secondary mutations allowing resistance to higher concentrations can be promoted by the SOS-response, which can modulate genetic instability and stimulate HGT that spreads the resistance mutation within the population [10]. In addition, bacteria can develop compensatory mutations that reduce the fitness cost that is usually associated with antibiotic resistance [11].

The molecular mechanisms of antibiotic resistances have recently been summarized by Blair et al. [3]. These include a prevention of the access of the drug to its target due to a decreased permeability or increased efflux. Furthermore, structural changes of the antibiotic target are able to circumvent an efficient binding of the antibiotic. This is caused by point mutations of genes coding for the antibiotic target, which will be rapidly selected under continuing antibiotic pressure. Targets can also be protected by mechanisms of resistance involving chemical modification, which can be induced by the antibiotic (and are therefore transient); however, mutations might produce a constitutive (permanent) expression of these mechanisms.

How a resistance mechanism works at the single cell level can affect its abundance in the population. It has been observed that so-called ’selfish’ mechanisms such as efflux-pump are selected at lower antibiotic concentrations than mechanisms that lead to antibiotic modification that also benefits non-resistant cells in the population [12]. Genes that are involved in the inactivation of antibiotics are sometimes colocalized with other genes that enable bacteria to use the antibiotic as a nutrient and energy source [13]. This exploitation of antibiotics as a resource in turn also benefits sensitive cells and adds another level of complexity to the process of antibiotic resistance proliferation.

Bacteria which do not harbour any or simply not the appropriate resistance are killed or do not replicate in the presence of antibiotics [14]. The effect of an antibacterial agent can differ between species or strains of the same species. A bacteriostatic effect is given when a drug (most of all) inhibits bacterial growth, e.g. by blocking a specific metabolic pathway, and affected bacteria may resume their growth if the antibacterial agent gets diluted [15]. Bactericidal effects are given if a drug is able to kill the bacteria at high rates, which is mediated by an irreversible destruction of the physical or genetic integrity of individual cells and finally leads to a more or less pronounced decline in the number of viable cells [14]. However, below the minimum bactericidal concentration (MBC), they may only inhibit bacterial growth. Bactericidal effects of antibiotics can be undesirable in certain clinical settings, e.g. when cell lysis releases bacterial products that stimulate the production of other substances which may initiate a harmful inflammation [15]. Meta-analysis studies have shown that the clinical outcome (even in serious infections) using static or cidal drugs is not substantially different, and probably static agents can act sometimes as cidal and viceversa in the complex environment of the infection site [16]. The antibiotic effect thus depends on the general effect of a drug on a target bacterium and on the antibiotic concentration or its dynamics.

Antibiotics used for therapeutic treatment of human infections, in agriculture and animal or fish farming pollute the environment and act as a selective pressure for resistances, potentially increasing the risk of transfer of resistance genes to human pathogens [17]. For example, it has been observed that high background concentrations of quinolones in a river enriches the population of waterborne bacteria carrying plasmid-encoded quinolone resistances [18]. Additionally, heavy metal pollution is considered to be a selective force for antibiotic resistances, because a co-selection occurs by mechanisms such as efflux pumps that can work against both heavy metals and antibiotics (cross-resistance) or by a close genetic arrangement of both types of resistance, which facilitates a combined transmission by HGT (co-resistance) [19].

The transfer of resistances from their original (natural) hosts to human pathogens has been suggested to be restricted by stringent bottlenecks related to fitness costs, the founder effect and ecological connectivity [1], all of which also apply to plasmid-coded antibiotic resistance. Accordingly, transfer events require a contact between recipients and donors, implying that both the size and connectivity of their populations in the same or adjacent ecological space are important. Where the background concentration of antibiotics is very low, cells carrying a resistance plasmid may have a lower fitness and may only be maintained by the acquisition of compensatory mutations or the connection to another habitat with different conditions, which has been recently demonstrated by an experimental evolution study for the maintenance of a mercury resistance plasmid [20]. Martinez et al. [1] further assumed that the chance that a new gene is established in a population is reduced when other genes with a similar substrate profile are already stably spread within this population (founder effect). Nonetheless, there can also be pleiotropic effects, as those recently observed for co-evolving plasmid-host pairs, which suggest that the stable presence of resistance plasmids can increase the stability of other resistance plasmids in the same host [21] – a mechanism that facilitates multidrug resistance. Hall et al. [22] observed that the fitness effect of plasmids was highly variable and depended on several factors including the plasmid status (of competing hosts) and the degree of abiotic selection of the plasmid-coded resistance. These considerations and empirical observations suggest that the proliferation of antibiotic resistance plasmids is likely to be influenced by multiple abiotic and biotic factors.

**Fig. 1:**
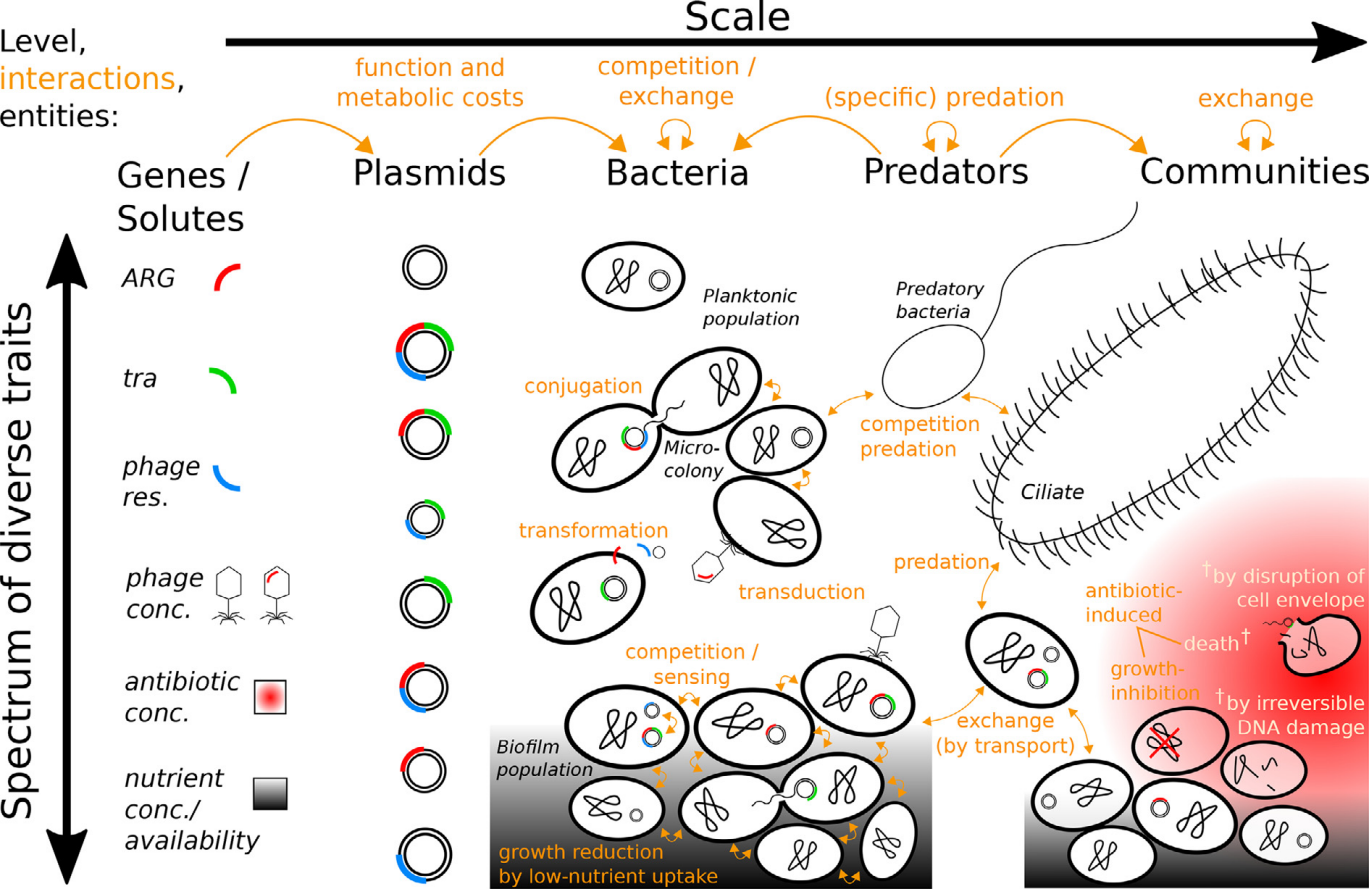
**Entities, state variables and scales affecting the ecology of plasmid-coded antibiotic resistance**. Plasmids vary in size and can provide antibiotic resistance genes (ARG) or phage resistance to their host. This can help cells to prevent growth inhibition or cell death in consequence of the presence of some antibiotic. If plasmids carry transfer genes (tra), they can perform conjugation, which transfers a copy of the plasmid from the host to a nearby recipient cell. Bacteria can be either solitary or part of a microcolony or biofilm. Those that detach from the biofilm may be transported to another location where they can exchange genes with other subpopulations or members of the microbial community.

Martinez et al. [23] proposed some guidelines that were drawn up to predict the risk of emergence of resistances to antibiotics which will become clinically introduced. Accordingly, a prediction of the dissemination of antibiotic resistances should take into account evolutionary constraints, selection pressures and environmental variations. To assess the probability that resistant bacteria become established these authors recommend developing mathematical models which investigate the population dynamics of resistant and sensitive bacterial populations in the absence and presence of antibiotics. In general, a variety of factors is at play, and selection effects can occur for different targets nested at different scales, including landscapes, soil particles, biofilms, bacterial populations as well as the bacterial host, MGE, and the resistance gene itself [24]. For the ecology of plasmid-coded antibiotic resistance, certain entities, state variables and scales are particular important (Fig. 1).

In the following, processes that influence plasmid propagation and the characteristics of diverse aquatic environments are reviewed to provide an overview of the complexity that models on the spread of plasmid-bornantibiotic resistances are confronted with. Finally, this overview provides an insight into opportunities for the application of different types of simulation models and opens an ecological perspective for plasmid and antibiotic resistance research.

## 2 Plasmid fitness

**Fig. 2:**
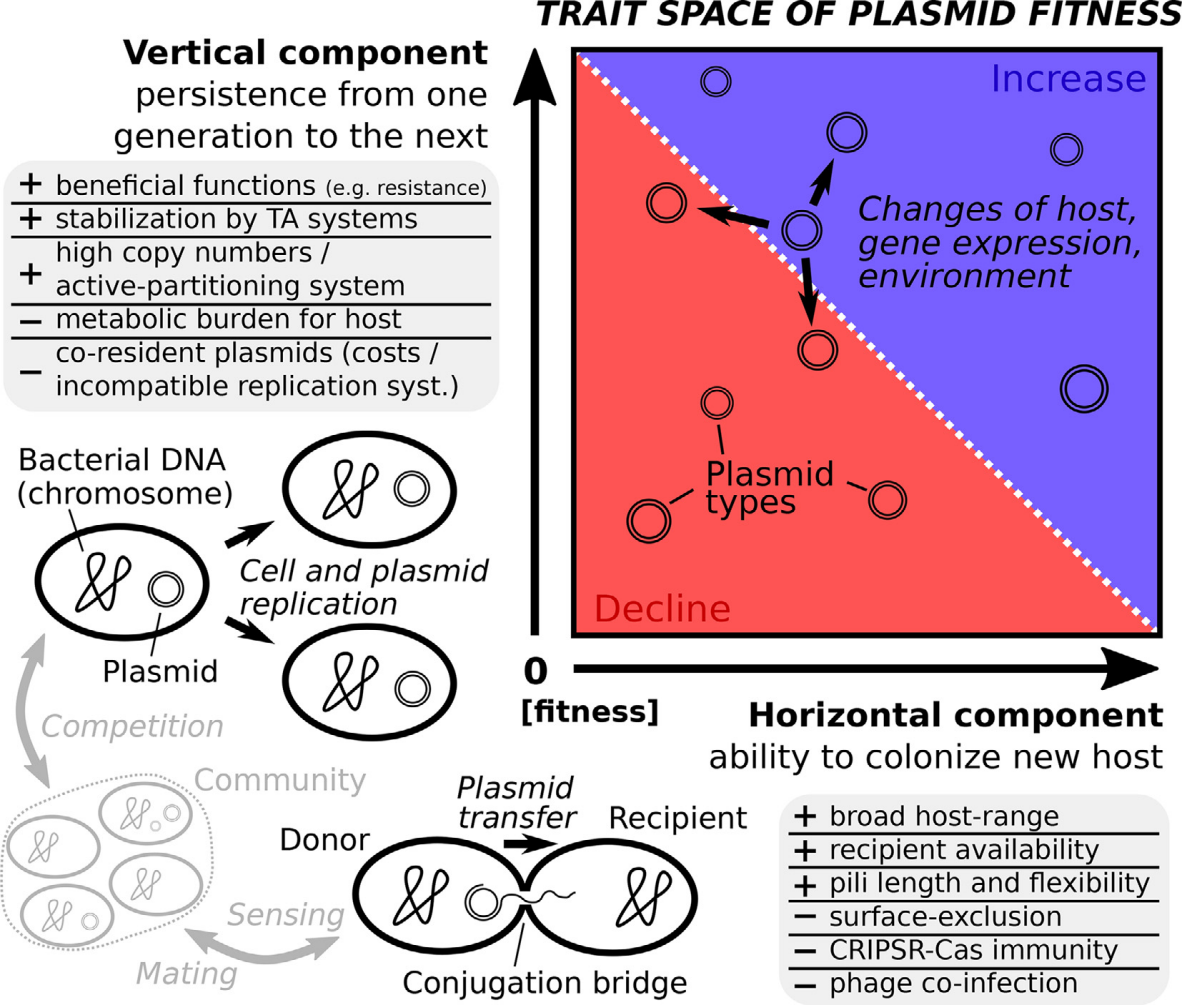
**Trait space depicting the potential realized fitness according to a plasmids vertical and horizontal transmission fitness.** Its frequency in a local community declines if vertical transmission fitness is low, e.g. due to high rates of missegregation and plasmid costs, and if this cannot be compensated by horizontal transmission, e.g. conjugation. Both vertical and horizontal transmission fitness are not constant for a specific plasmid, but can be host-specific and change over time due to evolutionary modifications and spatio-temporal variations of environmental factors (see main text).

Fitness can be considered as a measure of ecological success representing the ability to leave offspring relative to others [25]. In this context, the fitness of plasmids has two dimensions (Fig. 2): vertical transmission fitness, i.e. spread to the daughter cells of the host by cell division, and horizontal transmission fitness, i.e. spread to new host cells, for example, by conjugation. Considering that any effects that reduce vertical plasmid transmission can theoretically be balanced by the effect of horizontal transmission, the conditions for plasmid persistence can be analytically determined 26, [27], [28], [29], [30]. This can be easily generalized to a specific plasmid in specific and axenic bacterial isolates (clones) and has provided important insights into plasmid dynamics. However, apart from such a ’subpopulation-centric-framework’ [31], bacteria in nature often live in complex communities (as illustrated in Fig. 1) and dynamic changes in the rates of vertical and horizontal transmission are to be expected because (i) different bacteria may be available as hosts, (ii) they also compete with each other, and (iii) may carry other plasmids, (iv) all of which can undergo evolutionary modifications and (v) are subject to changes of environmental conditions. As current research aims to understand the environmental dimension of antibiotic resistance, such ecological and evolutionary factors have to be taken into account.

### 2.1 Vertical transmission

Particularly important for understanding the persistence of antibiotic resistance is the general question how plasmids that provide no apparent benefit persist in a bacterial community. On the one hand, the persistence potential of a specific plasmid type might be increased if it is able to replicate in multiple hosts and has a less severe impact on the growth rate of the plasmid-bearing host in comparison to its plasmid-free counterpart. For example, Seoane et al. [32] reported that plasmids can reduce maximum growth rates by up to 40% compared to the plasmid-free state. On the other hand, plasmids benefit if they secure their own propagation during cell fission. This can be achieved in different ways: (i) high copy numbers can reduce the rates of missegregation, i.e. that a daughter cell receives no plasmid copy, (ii) an active-partitioning system can control successful segregation of the plasmid copies, (iii) the so called ’post-segregational killing’ by a toxin-antitoxin (TA) system rapidly, but reversibly reduces the growth of daughter cells that do not receive a plasmid copy (which encodes the ’antitoxin’ that acts like a silencer of the growth inhibitor; TA systems do not kill bacteria [33]). The latter might prevent that plasmid-bearing cells get outcompeted in the absence of positive selection for plasmid borne traits [34]. However, this may not efficiently work when a population consists of a number of bacteria (of other species) that are either relatively or completely immune to it or that just never receive the TA system through horizontal transmission and are therefore not affected by it within their cellular lineage.

### 2.2 Horizontal transmission

**Fig. 3:**
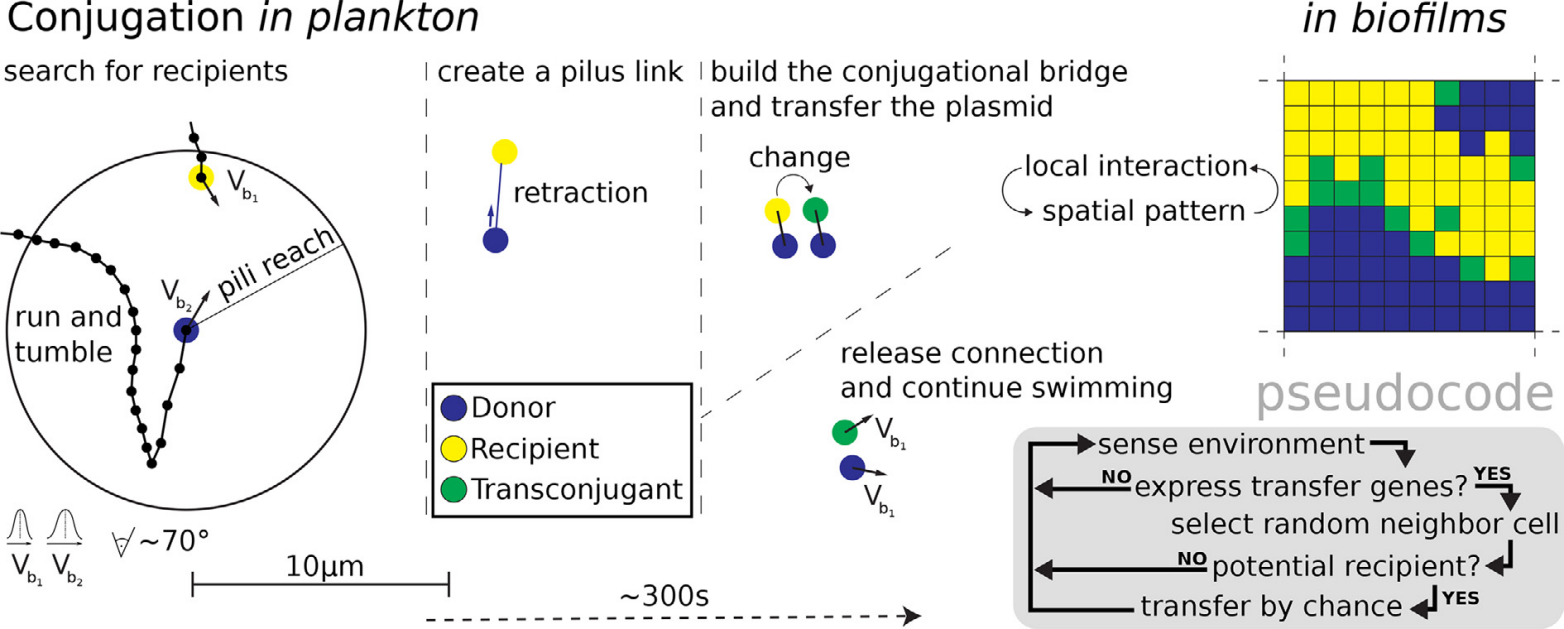
**Scheme of horizontal gene transfer by mating pair formation of bacteria in a planktonic environment:** a bacterium that harbors a conjugative plasmid, called ’donor’, couples through its pilus to another bacterium that will receive the plasmid, called ’recipient’. When a copy of the plasmid was successfully transferred, the recipient finally becomes a new donor, called transconjugant. $V_{b}$ denotes the velocity at which bacteria swim in a planktonic environment, either before ($V_{b_{1}}$) or after a tumble event ($V_{b_{2}}$) (for a short time) by which bacteria change their movement direction. In a biofilm, local interactions occur without further motion of individual cells (colored patches/pixel). This can create spatial patterns that in turn affect how and which bacteria interact with each other. The pseudocode gives an example for a conjugation submodel that considers sensing, adaptation and stochastic local interactions of individual cells. This may also be applied to conjugation in plankton, when only mixing but not swimming of individual cells is considered.

Autonomous replication is common to all plasmid types, but further capabilities can vary remarkably. One of the most important differences is their ability to perform horizontal gene transfer, which is known since the pioneering work of Lederberg and Tatum [35]. Plasmid types that encode a set of mobility genes (MOB) as well as a complex enabling mating pair formation (MPF) are called ’conjugative’ [36]. They can spread from one bacterium to another through a mating channel, provided by a type 4 secretion system (T4SS) [37]. The scheme provided in Fig. 3 illustrates this process: the bacterium that carries the conjugative plasmid expresses one or more pili. These are extended to capture another bacterium and, if successful, retracted in order to minimize cell distances. This establishes a stable cell-to-cell contact for the transfer of a plasmid copy to the recipient, which in turn becomes a transconjugant. The complete transfer process might take some minutes, as it was shown in detail for individual mating pairs by live cell imaging [38], extending the first information presented decades ago [39].

Plasmid types are called ’mobilizable’, if they encode the mobility genes, but only hitchhike with the MPF system of other co-occurring conjugative plasmids performing conjugation [36]. This can also occur in the opposite direction, i.e. from the receiving to the donoring cell of the conjugative plasmid, called ’retro-transfer’ [40].

Plasmid types that are neither conjugative nor mobilizable are called ’nonmobilizable’ or ’non-transmissible’ [36]. They could also be horizontally transferred by natural transformation, transduction or conduction [41], which refers to the uptake of DNA from the environment, the dissemination by phages and the co-integration with a transmissible plasmid. However, the rates of these processes are considered so low that most authors describe this type of plasmid mobility as ’non-transmissible’ [42], [41]. Therefore, non-transmissible plasmids are restricted to spread by cell fission, i.e. vertical gene transfer (VGT). Smillie et al. [36] and Garcillan-Barcia et al. [41] found that globally a fourth of the plasmid types are conjugative, one fourth mobilizable and a half non-transmissible.

### 2.3 Plasmid co-occurrence, incompatibility and immunity

Different plasmid types can co-occur within the same cell, but if they share one or more elements of the plasmid replication or partitioning system, this will likely lead to increased segregation rates, making these plasmids incompatible to each other - a feature that is in general describing the failure of two co-resident plasmids to be stably inherited in the absence of external selection [43]. Thus, related plasmids belong to the same incompatibility group, which means that the introduction of one of both destabilises the other. Despite this, incompatibility can also work through horizontal transmission, since specific plasmid-coded entry exclusion systems let cells actively prevent the transfer of incoming plasmids belonging to the same or a related incompatibility group (e.g. IncC and IncA) [44]. This happens by the recognition of proteins involved in conjugation that are specific to the respective incompatibility groups.

Recent experimental studies demonstrated that the co-occurrence of plasmids affects the fitness of a single plasmid. On the one hand plasmid survival can be promoted by positive epistasis [45], which refers to decreased costs of co-residing plasmids in comparison to a cost that might be expected when their single costs are summed up. On the other hand, epistasis can also be negative or neutral. It has also been reported that horizontal transfer rates are often decreased, when two [46] or three [47] distinct plasmids interact.

Bacteria can be immune to an incoming plasmid due to clustered, regularly interspaced short palindromic repeat (CRISPR)-Cas systems, which have been demonstrated to prevent conjugation [48]. In a recent study, Pinilla-Redondo et al. [49] showed that these system are carried by plasmid-like elements and involved in competition between plasmids. These authors concluded that they are used to eliminate other plasmids with similar properties and lifestyles in order to monopolize the host environment. This indicates an important role of these systems for the regulation of horizontal plasmid transfer between microorganisms. However, CRISPR systems are highly diverse and the reasons for this diversification and its consequences for the evolution and genetic adaptation of microbial communities are not yet fully understood [50].

### 2.4 Plasmid costs

It is widely accepted that the main fitness cost of plasmids comes from downstream events such as expression of plasmid genes [51], although plasmids also incur costs for DNA replication and repair. The production of plasmid proteins, e.g. for conjugation, uses up raw material and occupies the cellular machinery [7]. Several studies identified conjugation as a source of plasmid cost [51]. Considering that most bacteria suppress the expression of transfer genes [52], this is probably a strategy to avoid ongoing high costs. In contrast to this mechanism, it was found that functions such as antibiotic resistance may not be regulated in response to environmental conditions, i.e. may incur costs even in the absence of antibiotics [53].

### 2.5 Adaptations at population and cell level

The spectrum of actions bacteria are able to perform under suitable conditions is restricted by the genetic information they have access to. A number of mechanisms such as point mutations, deletions of chromosomal regions as well as processes procuring DNA from the environment can create novel genetic variants in bacterial populations [25]. In addition to the stable core genome of around 2000 genes for a single E. coli cell (assessed for 20 E. coli strains) [54], bacteria gain and loss genes which are highly mobile between bacterial cells [55], e.g. by conjugation. These non-core genes increase the diversity of a species pan-genome and can make up 90% of it [54]. Hence a bacterial population has a fundamental basis to adapt to various environmental requirements. But these shifts on population level are subjected to adaptations of individual cells. The part of a bacterial genome that encodes for additional traits that are only sometimes beneficial, but do not represent essential cellular functions, is called the ’flexible’ gene pool [5].

If ecological conditions become harsh bacteria may adapt through alterations of their genome structure. This happens by the selection of point mutations and genetic rearrangements as well as by gene acquisition through HGT or by genome reduction [5]. It results rather in modifications of the ’flexible’ gene pool, which comprises variable chromosomal regions as well as mobile and accessory genetic elements, than in alterations of the conserved ’core’ genome, encoding essential cellular functions [5]. The latter mostly emerge through recombination and mutation [54].

Mobile genetic elements such as plasmids may provide advantageous traits to the host, potentially increasing its fitness. Those bacteria possessing highest compatibility and performance in interaction with these MGE will presumably be most competitive [1]. If compatibility is low or the carriage of the MGE in its ancestral form is to expensive compared to its advantages, adaptation has to occur. Otherwise the integrated MGE, the bacterium or both will get lost, at least in the long-term. This becomes evident to a greater extend if the conditions for the selection of the trait are fluctuating or become obsolete and the carriage of these genes represents a competitive burden for the bacteria. In the study of San Millan et al. [42] both a reduced cost and periods of positive selection were necessary to maintain the integrated MGE, a non-transmissible plasmid. Environmental conditions, starvation, stress and high copy numbers of a gene increase the probability for genetic mutations, which could be important in the evolution of antibiotic resistances [23]. Such mutations may alter the biological fitness of resistant strains to a level similar or even higher than that of the susceptible parental strain.

Many studies investigated the coevolution of plasmids and bacteria. It was found that the plasmids cost-of-carriage can be reduced due to mutations occurring at the plasmid, the chromosome or both [7]. Among the key mechanisms that mediate this amelioration of plasmid costs are changes in the conjugation rate, the loss of plasmid genes as well as changes in plasmid gene expression [7]. Changes in conjugation rate can range from a complete loss of the ability to conjugate towards the evolution of lower or higher rates [56], whereas the imposed costs to the host positively correlate with the extent of the conjugation rate [34]. Hence, a shift towards a lower rate enables a higher vertical transmission rate, which indicates a closer alignment of plasmid and bacterial fitness interests. Dahlberg and Chao [56] reported deletions of plasmid-borne antibiotic resistance genes in plasmid-containing populations during experimental evolution. This resulted in an increased fitness of the host, but also means that the resulting population is again sensitive to antibiotics.

As plasmid-encoded compensatory adaptations are not only confined to one population as adaptations of the bacterial chromosome, their potential to invade new populations by horizontal gene transfer may be enhanced, as recently demonstrated by mathematical modeling [30]. Dionisio et al. [57] found that plasmid adaptations can provide a general fitness improvement, even in alternative hosts. This enhances the potential for plasmid spread and maintenance dramatically, even when it was reported to be very low for a specific plasmid-host-combination. This finding is of particular importance considering that recent bioinformatic analysis revealed that more than 60% of the plasmids of the global prokaryotic plasmidome were reported to be able to colonize species from different phyla [58]. Plasmids coding for antibiotic resistance could therefore initially acquire cost-compensating mutations in co-evolution with a particular common host and later spread as optimized vectors to other less common but potentially more harmful species.

**Fig. 4:**
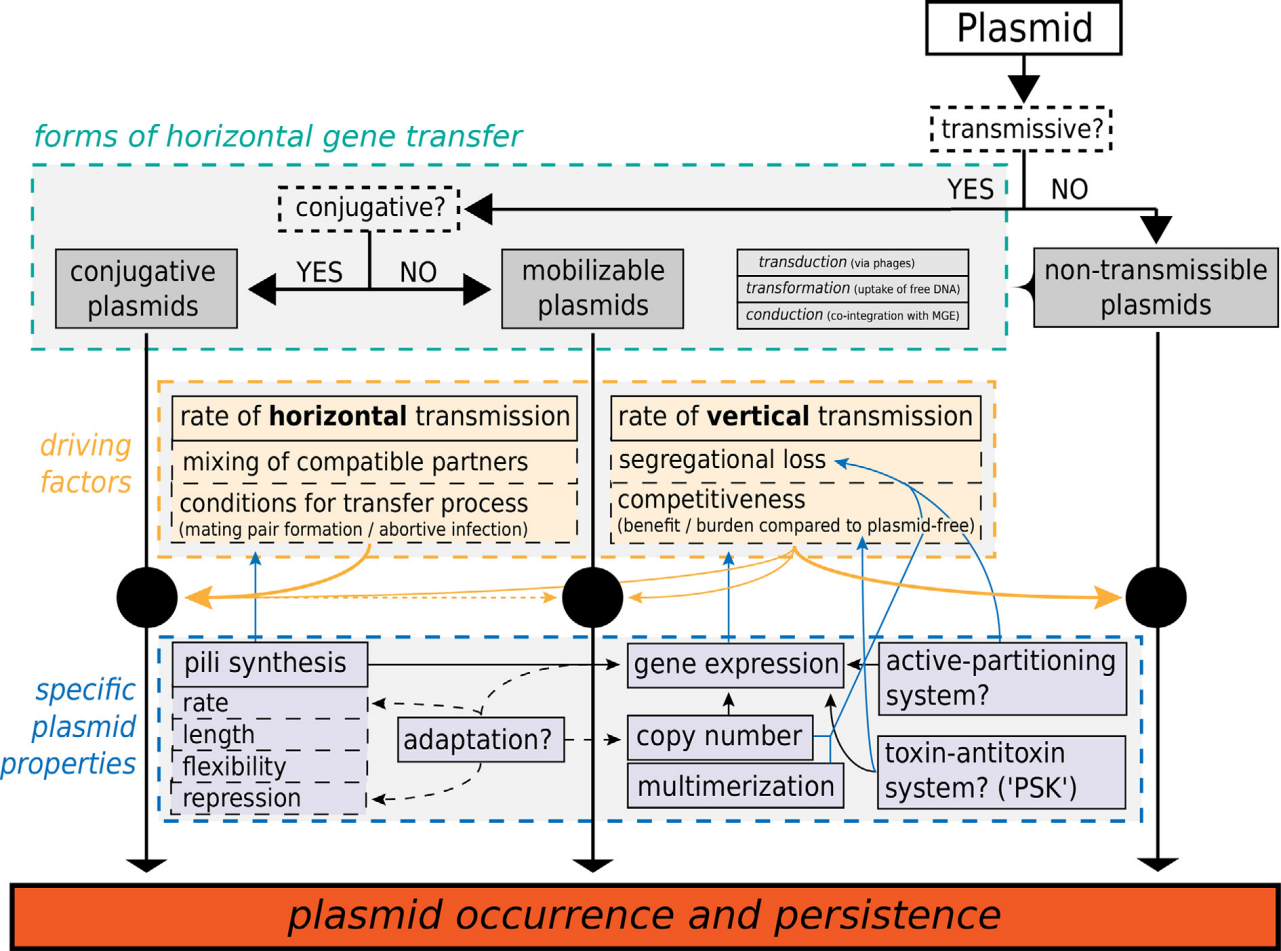
**Properties of different plasmid types and influences on their vertical and horizontal transfer capabilities**. These can also determine the spread of antibiotic resistance. Apart from the factors described here, many other ecological conditions are likely to play an important role (see main text).

The spectrum of all the aforementioned properties and processes shows that the occurrence and persistence of plasmids, which may provide antibiotic resistance, is affected by diverse factors comprising mutations and adaptations at the single cell level as well as biotic interactions between different plasmid types and different plasmid-host associations. Fig. 4 attempts to summarize how certain properties of different plasmid types determine the propagation of plasmids and how this can be influenced by different processes. The following section focuses on the role of environmental and ecological factors.

## 3 Environmental characteristics

### 3.1 Biofilm versus plankton

Microbial processes and all kind of ecological interactions greatly depend on the features of the environment. In general, bacteria living in aquatic environments are either in a planktonic status or they form a biofilm, a well organized structure of microorganisms that is protected by a self-produced polymeric matrix. A biofilm can be attached to a fixed surface or to another biotic or abiotic particle that itself might be dispersed in the free water column. Both modes, the planktonic and the biofilm mode of bacterial life, are schematically illustrated and compared in Fig. 1 and 3. Biofilm-formation is a very common and successful strategy employed by bacteria, whereas timescales differ between bacterial species and have been reported to take as long as 20 to 25 days for maturation [59]. Biofilms play a significant role in microbial survival [60] and mediate the vast majority of healthcare associated infections [61]. However, bacterial populations also develop in situations in which individual cells are not interconnected, and all bacteria found to be able to form biofilms can produce ’free-swimming’ cells.

In plankton, the bacteria are mixed and have many opportunities for horizontal gene transfer through mating pair formation, while the total cell density is usually reduced by a low nutrient concentration and a high influence of dilution or washout, as is typical in a river. Toxin-delivery systems that can influence the composition of microbial communities and processes such as the secretion of virulence factors or the production of public goods that are initiated by cell communication might not be at work under such circumstances. Quorum sensing mechanisms usually prevent actions that would be unproductive and costly when undertaken by only a very few cells [62]. However, bacteria are often capable of taxis and can aggregate by this means to increasing local concentrations, or by associating with fibres and particulates, e.g. similar to activated sludge flocs in a water treatment reactor. The formation of such microcolonies that are dispersed within the water column may also stimulate horizontal gene transfer systems that allow bacteria to sense the right time and place, e.g. high cell densities, to switch on the conjugation machinery [52].

Opposed to this, bacteria in a biofilm predominantly interact with their local neighbors, which can enhance the efficiency of the before mentioned mechanisms. The same is true for other mechanisms that require cell-to-cell contact, such as contact dependent inhibition (CDI), a competitive system that can influence the composition and spatial organization of growing bacterial populations [63]. Such an antagonistic behavior based on the ability to kill or inhibit the growth of other bacteria has also been observed to evolve in stationary phase [64], i.e. when the population stops growing. Considering the high cell density and potential huge distance to the nutrient supplying interface, which is either the surface the biofilm is attached to or the fluid flowing over and through the biofilm matrix, bacteria in a biofilm likely face heterogeneous environmental conditions depending on their (vertical) position within the biofilm matrix [65]. This biofilm matrix is of particular importance because it can represent a barrier for antimicrobial agents, which, in contrast to the high importance of biofilms for bacterial life, have generally been tested considering how they eradicate planktonic bacteria [66]. Due to the impermeability characteristics, a higher concentration of antibiotics may be required to have a bactericidal effect on biofilm bacteria compared to planktonic bacteria [67]. This can facilitate the survival of persister cells, which appear for several reasons, but may also survived a previous antibiotic treatment and can easily provide resistance to the regrowing population [68]. New therapeutic approaches therefore target biofilm formation and dispersal [66].

At the interface of biofilm and plankton, bacteria may either attach from the plankton to the biofilm, which can be promoted by the adhesive effect of pili synthesised by cells carrying conjugative plasmids [69], or otherwise be dispersed from the biofilm to the plankton, which can be modulated by quorum sensing of diffusible signal factors [70]. Recently, the ability of conjugative plasmids to promote biofilm formation has been found to be correlated with conjugation efficiency, whereas some plasmids can have dominant effects over other co-inhabiting plasmids [71]. Biofilms grow because cells divide and shove each other away, a mechanism vividly demonstrated by individual-based models of colony formation [72] Apart from this effect, colonising planktonic cells likely remain in the same location until they either die or re-enter the planktonic phase [73]. This is likely associated with a higher degree of intraspecific competition compared to a mixed environment, where bacteria are less likely to interact directly with their progeny.

Biofilms are thought to be highly heterogeneous with respect to many environmental conditions such as nutrients, pH and oxygen gradients, as well as the metabolic potential and phenotypic characteristics of the constituent populations [74]. The niche space, which is typically characterized by time, space, resources and predation [75], could therefore also be considerably large from the plasmids’ point of view. Correspondingly, it was hypothesized that HGT could play a key role in microbial sociality in a biofilm context, since many accessory features of the plasmid are expressed outside the cell [7]. This may also explain why multispecies biofilms have been found to show increasing resistance [60]. Nonetheless, another recent study reported a reduced selection for antimicrobial resistance when embedded in a natural community [76], which indicates that still much is unknown about the role of interspecific interactions.

### 3.2 Natural versus artificial conditions

One of the differences between artificial and natural aquatic environments is their pollution with antibiotics, which is either increased by direct entries into sewage systems and waste water treatment plants or rests at a low natural background concentration as in almost pristine rivers. However, antibiotic resistance genes have been found in any type of aquatic environment, even in drinking water [77]. In the Danube river, one resistant isolate could be detected for each tested antibiotic, which also opens the possibility that these genes can spread to related human pathogens [78]. In India, extended-spectrum β-lactamases are known to circulate in the community, although affected people may not even have been hospitalized in the past - a problem that has also been linked to antibiotic resistance found in tap water and street water basins [79]. It indicates that pollution with antibiotics is concerning at both artificial and natural environments because they are interlinked and even water cleaned in waste water treatment plants can spread antibiotic resistance into the environment, where it may re-enter the drinking water cycle.

To understand the environmental dimension of antibiotic resistance, it is of particular importance to be aware of the differences between the artificial conditions in the laboratory and the natural conditions in the environment. In general, these have a biotic and an abiotic dimension resulting from the targeted homogenization and determination of many factors influencing bacterial life in the laboratory and their heterogeneity and dynamics in the natural environment. Among the biotic factors is biodiversity, which is linked to a consideration of intra- and interspecific interactions of bacteria and their interactions with entities of other trophic levels such as plasmids, phages and predators. These entities and their interactions can further be affected by demographic and spatio-temporal dependencies such as those given by a restricted spatial interaction in biofilms or by rare mutations and other events that happen at the single cell level as a result of a specific life history. The abiotic dimension comprises the diversity and spatio-temporal variation of the physico-chemical conditions, given by local temporal variations in the availability or presence of specific substances such as antibiotics, heavy metals, organic compounds, oxygen or derivatives of bacterial processes, as well as by the local temperature and dilution regime, which potentially influence each other in their dynamics.

Testing ecological and evolutionary theory with simple, highly controlled laboratory setups with one or two species provides very important insights, but several characteristics can be strongly altered when the biotic complexity is expanded [80]. Even when simple setups are used, conventional laboratory cultivation techniques may fail to appropriately reproduce the natural conditions of organisms [81]. Whereas closed batch cultures impose a drastic change of the environmental conditions during the experiment, open continuous-cultures fix these conditions but may also neglect that the growth of heterotrophic microorganisms is limited in most environments by the slow hydrolysis of particulate organic matter [81]. The doubling times of bacteria are substantially longer in the wild than in the laboratory, e.g. 15 h instead of 20 min for *E. coli* [82]. Although environmental matrices can be maintained in a laboratory context, the microbial communities may behave and evolve differently than in their natural equivalent [83]. Recent methodological advances providing synthetic bacterial communities comprising for example 33 bacterial strains bridge the gap between simpler synthetic and natural systems [80]. Other developments such as community flow cytometry, which provides high-dimensional data characterising communities at the single cell level [84], can enhance the analysis depth and resolution of sequencing approaches and can be used for pure cultures or complex communities in clear medium as well as challenging matrices [85]. The development of such approaches can overcome many restrictions, for example of simple laboratory models focusing on single-species biofilms, which do not illustrate the true nature of biofilm communities and ignore that many if not most infections are mediated by polymicrobial biofilms [60].

It was also found that regular switching of the plasmid host is able to increase the amelioration of the plasmids cost-of-carriage compared to plasmid evolution in a single host species [86]. Similar findings have also been reported from soil microcosm experiments [87], suggesting an increased potential for amelioration of plasmid costs in more complex (natural) environments compared to laboratory conditions, which may raise general concerns about their validity. However, Hall et al. [88] found that natural communities might even not adapt to abiotic conditions, because such fitness benefits can be negated considering competitive species interactions in natural communities. Similarly, Cairns et al. [89] showed that a plasmid-dependent bacteriophage can eliminate a conjugative plasmid providing antibiotic resistance, but this can be prevented by a protozoan predator. It suggests that multitrophic interactions play a significant role, although many experimental setups do not take this into account.

## 4 Modeling approaches

### 4.1 Purpose of models

Just as laboratory systems do not reflect the actual conditions in nature, simulation models represent a simplification of the depicted system. Thus, if the depcited system is a simplification, many simulation models represent a kind of simplification of a simplification. This is because any experimental design is guided by an *implicit* model, an imagination of the system under investigation, but which lacks a clear definition of assumptions and tests of consistency and logical consequences [90]. This represents a form of ’pre-model scaling’ [91], a simplification or aggregation that is done prior to running ’a model’. Usually, people have an *explicit* model in mind, if they think of ’models’ or ’modeling’. Contrary to *implicit* models, the assumptions of *explicit* models are laid down in detail. It can be studied what happens on the basis of these assumptions and what happens if they are changed. Writing such *explicit* models means that the results are replicable and can be subjected to rigorous sensitivity and uncertainty analysis.

### 4.2 Theoretical versus customized models

As models thus offer a gain in knowledge from the abstraction of the represented real system, it is important to be clear which system the model actually represents. A distinction can be made between two types of simulation models: 1) theoretical models that are guided by a specific scientific question or theory and are primarily developed to test contrasting hypothesis; and 2) customized models that are guided by a specific experimental design and are primarily developed to serve as a tool for the analysis of the experimentally observed behavior (data). Both type of models can provide mechanistic insights that cannot be revealed by laboratory experiments. While ’customized models’ show how the experimentally observed patterns can arise, ’theoretical models’ are not constrained by the design of a laboratory experiment. They can even address issues that cannot be investigated by experiments. This is particularly important for research in areas outside microbiology, as it takes much longer to experimentally observe, for example, evolution and ecological interactions in forests. However, also in microbial ecology there are many interesting setups that are hardly implementable. These can be addressed by theoretical models.

#### 4.2.1 Theoretical models

Theoretical models are often more flexibly applicable than similar customized models because they provide a more general description of a system. One such example is the platform iDynoMiCS (individual-based Dynamics of Microbial Communities Simulator) [92], which is composed of modules, a feature that facilitates its application to varying settings. It has for example been used for theoretical studies addressing diverse topics such as the response of a community of denitrifying bacteria to an environmentally fluctuating oxygen availability [92], the dependence of conjugation on the growth rate of the donor cell and its consequences for the plasmid invasion in biofilms [93] as well as the effect of competitive and mutualistic interactions in a two-lineage community on the selection of antibiotic resistance [94]. Another recent modular platform that aims to serve as a kind of standard model is the Antibiotic Resistance Evolution Simulator (ARES), which can simulate nested compartments from the ecosystem level to the bacterial host [95]. It has been applied to examine how the rate of antibiotic resistance among bacterial species is influenced by a variety of variables that determine the complex parameter space that defines the interaction of biological elements in a given environment [96] and how the plasmid kinetic values that are determined by conjugation rate and segregation rate due to stochastic loss and incompatibility with other plasmids influences the population ecology of antibiotic resistance in a hospital setting [97].

Besides iDynoMiCS and ARES, many more theoretical models have been recently applied to study the spread of antibiotic resistance, e.g. to investigate the role of varying mechanisms to solve conflicts between plasmids carrying a cooperative trait and their bacterial hosts [98], of transmission and relatedness as factors driving the plasmid-borne public goods production [99], of multispecies interactions involving antibotic production and degredation on ecological stability and biodiversity [100], of inter- and intra-cellular interactions between conjugative and non-transmissible plasmids [101], of switching strategies between susceptible and persister cells [102], of plasmid- versus chromosome-located compensatory adaptations of the cost-of-carriage of plasmids and associated resistance genes [30], of the positioning of wastewater treatment plants in a river network [103], of transformation for a stabilization of the bacterial genome in stochastic environments [104] and of transduction considering co-evolution (phage immunity of bacterial hosts) and stochastic and local effects [105].

#### 4.2.2 Customized models

Among the more recent studies that used customized models are Yurtsev et al. [106], who investigated how the fraction of resistant cells carrying a plasmid that encodes a β-lactamase enzyme that may allow sensitive bacteria to survive is affected by antibiotic concentration and the addition of a commonly used β-lactamase inhibitor. For this, experimental results were compared with a model based on ordinary differential equations and a Michaelis–Menten Kinetics for antibiotic inactivation. A further example is the study by Lopatkin et al. [29], who derived a critical conjugation efficiency as an upper bound for dominant plasmid persistence and investigated the conditions of conjugation-assisted persistence when mutliple conjugation plasmids and multiple species are present. They used a system of differential equations whose increase in complexity was fully reported, starting with a one-species, one-plasmid model to a multiple species, multiple plasmid model. Malwade et al. [107] fitted varying systems of ordinary differential equations to flow cytometry observations of growth and conjugation dynamics in a batch process and compared the predictive power and parameter uncertainty of varying model formulations. Meredith et al. [108] quantified determinants of resistance at the single cell level and resilience at the population level in response to a β-lactam antibiotic. They also used a system of differential equations describing the interactive change of the components population density, antibiotic concentration (β-lactam), nutrient level and *Bla* concentration (β-lactamase). Valle et al. [109] studied the distribution of plasmid fitness effects for the major antibiotic resistance plasmid pOXA-48. They found this to be dominated by quasi-neutral effects, which, incorporated in a simple system of ordinary differential equations, indicated that plasmid stability increases with bacterial diversity and is less dependent on conjugation.

Further within–host and between–hosts models on antibiotic resistance dynamics including studies considering interactions with host immune responses or focusing on optimal drug dosage regimes were recently reviewed by Tetteh et al. [110]. Leclerc et al. [111] applied a systematic review on studies using ’dynamic’ models to study the horizontal transfer of antibiotic resistance genes between bacteria. They found that 33 of the 43 studies involved in their review used deterministic models to obtain their results, all of which representing ordinary differential equations (ODE). The other 10 studies used stochastic models, 6 of which representing agent-based models, 3 stochastic differential equations and 1 difference equations. The review by Leclerc et al. [111] further showed that nine of these studies did not apply any sensitivity analysis and only eight studies run their model multiple times and sampled parameter values from distributions rather than assuming them to be constant.

### 4.3 Population-level versus individual-based models

**Fig. 5:**
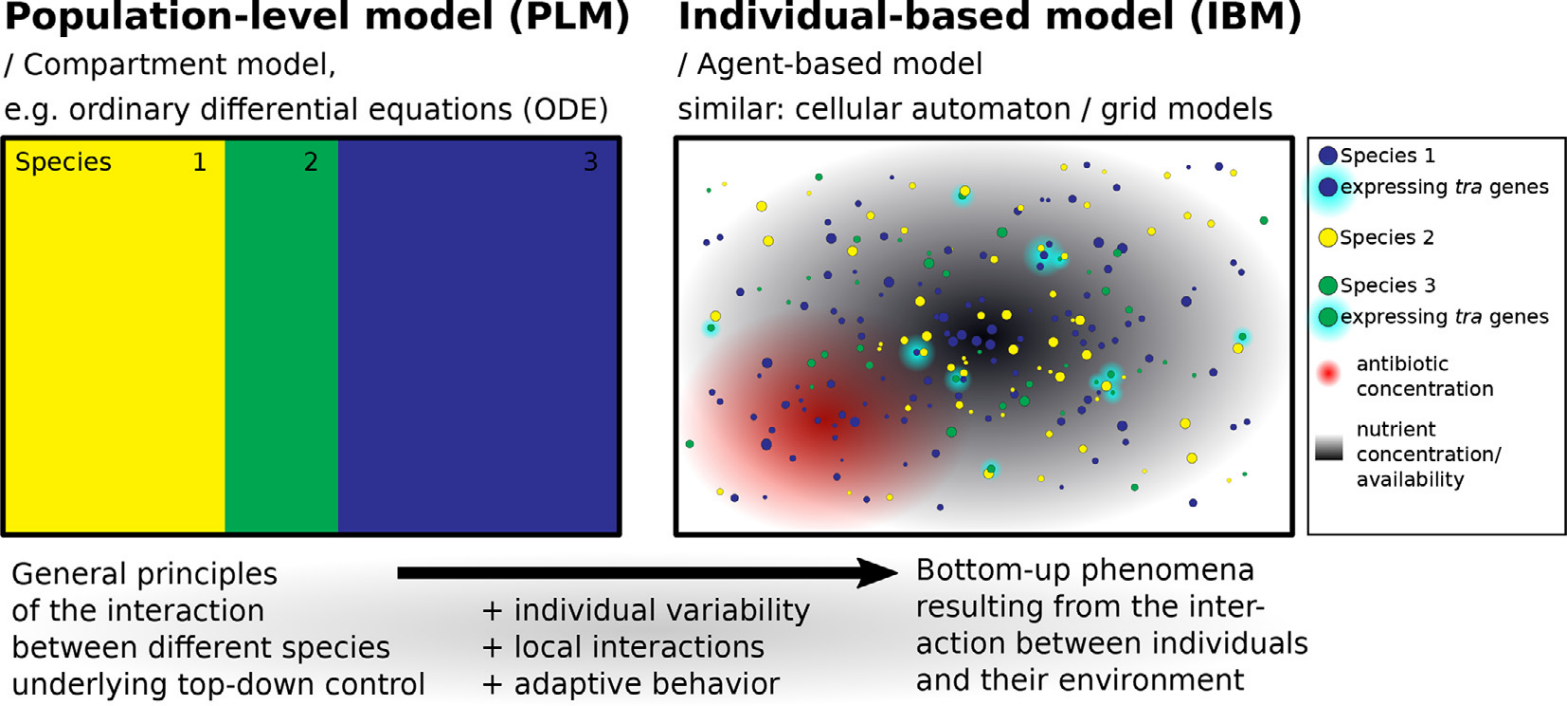
**Conceptual differences between population-level models and individual-based models.** Colors indicate different species that are represented either ’block’-wise (left; PLM) or by individual cells (right; IBM). The latter may also distinguish between cells of the same species in terms of location, biomass (size) and expression of transfer (*tra*) genes considering heterogeneous environmental conditions (with respect to nutrients and antibiotics).

Existing modeling approaches are differently suited to address certain research questions. A fundamental difference is the level at which these models describe interactions. On the one hand there are population-level models (PLM) that can describe the interactions between species. Ordinary differential equations (ODE) are often used to describe how a compartment of the model (e.g. one of the interacting species) changes over time. This is based on the principle of mass action, similar to chemical reactions. Thus, the degree of interaction or outcome of some process depends on the ’concentration’, ’proportion’ or ’density’ of the directly involved compartments. For example, horizontal gene transfer depends on the density of both plasmid-bearing and plasmid-free cells and a conjugation rate that determines how often they successfully encounter each other (and which is potentially subject to further restrictions; see below). If one imagines that the yellow compartment in Fig. 5 represents the density of plasmid-free cells and the blue compartment the density of plasmid-bearing cells, such an interaction might increase the area of the green compartment to the detriment of the yellow, considering that recipients become transconjugants as depicted in Fig. 3. The composition of the population of bacteria that live in a certain environment therefore changes. Such changes can also be resource-dependent, for example, by an explicit consideration that some nutrients are consumed, which control bacterial growth through some Monod-kinetic. A resource dependency can also be described by a logistic growth function that implicitly considers a decreased resource availability when population density increases. As an illustrative example, the black box surrounding the coloured compartments in Fig. 5 might be seen as the maximal cell density. Bacterial growth is the greater the more open (non-colored) space is left in this box. If it is full, as given, the density of bacteria cannot increase, only their proportions can change. Apart from that, some bacteria may die off over time, which means there is always some space left to fill by an increase of the compartments. The cell density of the total population or the single compartments could be theoretically infinitely small or large. Any change that can be observed at the population level arises due to the definitions that are directly described at the population level. This represents a ’top-down’ approach, since the observed behavior is a direct result of the model formulations.

Individual-based models (IBM) represent a fundamentally different approach. They often consists of (1) some individuals (or ’agents’) that interact with the world around them and/or with each other and (2) the world in which the individuals ’live’ or move around. Usually some rules define what every individual is allowed or has to do [112]. These rules are repeatedly applied in form of a loop, which allows to repeatedly act or interact.

IBM represent a ‘bottom-up’ approach, since the changes that are observed at the population level emerge from the activities of lower-level entities. For example, instead of describing the growth of an entire bacterial population, it is considered how individual bacteria take up resources, grow and perform cell fission. This also leads to population growth, but population growth as such is not part of the model formulation. Instead, such characteristics of the population emerge from the behavior of the individuals, which is determined by some rules. This behavior can also take into account individual heterogeneity, e.g. that individual bacteria, even those of the same species (which would belong to the same compartment), have different growth rates or perform cell division at different cell sizes. This may also reflect differences in their life cycle that themselves may be important for other processes, such as the ability to perform horizontal gene transfer [113].

An IBM might also consider that individuals adapt their behavior in order to understand and predict ecosystem complexities [114]. Bacteria can, for example, change their movement direction according to some chemical signal [115]. Thus, bacteria might direct their movement towards higher nutrient availability. Another example are bacteria that carry plasmid-bearing cells, but only shut off the repression of *tra* genes (means they become transfer-competent), when they sense that the conditions to transfer the plasmid are optimal [52]. One of the first real IBMs for microbial biofilms is ’BacSim’ [72], which assumes, for example, that bacteria perceive local densities and shove each other away, allowing them to remain competitive for the uptake of local resources.

The ability of IBM to be spatially explicit can be advantageous for a description of conjugation. An individual plasmid-bearing bacterium might therefore be able to perform a transfer attempt only to bacteria in its local neighborhood (see Fig. 3 and Fig. 4). Otherwise, it could also be sufficiently detailed to consider that individual bacteria encounter each other randomly in a mixed environment and perform conjugation with a certain probability. Besides conjugation, processes such as consumption of nutrients can be considered to occur locally, which means that bacteria directly compete for resources in the local neighborhood. Such differences in space can also be taken into account by spatially-explicit PLM, i.e. partial differential equations (PDE). This opens the possibility to compare different kind of models in order to reveal the effect of spatial structure (comparing ODE with PDE) or the effect of individual heterogeneity and adaptive behavior (comparing ODE with non-spatial IBM or PDE with spatial IBM), which is best practice [116] and has been recently applied unraveling the effect of spatial interaction on the evolution of a gene sharing mechanism [117].

New laboratory methods that enable to observe and measure the behavior of individual microbes also stimulates individual-based modeling [118], since data on the individual-level is required to parameterize an IBM and to compare simulated individual-level characteristics with empirical data on the same scale [116]. Various techniques extract and process information from individual microbes [119]. Cell tracking, for example, enables the analysis of cell trajectories that can be used to generate a distribution of the turning angle and the swimming speed (before and after a turning event) of bacteria [120]. Single cell analysis has been used to estimate the probability of successful conjugation in dependence to the donor-recipient orientation as well as distributions for the time required to transfer the plasmid and the delay time between transfer events depending on the individual cells history [113]. The application of scanning confocal laser microscopy of fluorescent bacteria revealed the dynamics of the spatial distribution of transconjugants in a flow chamber biofilm [40]. Cytometric analysis can sort thousands of cells in a second [121]. This can, for example, be used to determine relative abundances of subcommunities, reveal cell cycle dynamics and to monitor the evolution of microbial communities [85]. This opens various opportunities for the field of microbial individual-based ecology, which refers to the study of microbial ecology by the combination of such experimental data with individual-based models [118].

### 4.4 Limitations, pitfalls and good modeling practice

A variety of limitations of IBMs should be considered [116]. For example, IBM might be used to study the sources of variability between replicates of the same treatment in controlled laboratory experiments, as e.g. Harrison et al. [122] demonstrated. But when it comes to rare events such as an infinitesimal small probability that a specific mutations is acquired, IBMs might be on their limit, since they can only simulate a restricted number of individuals, but the probability that any of them will acquire such a mutation within a certain time depends on the population size. In such a case PLM might be required to perform simulation experiments efficiently, although they may only estimate the mean point in time when such a sweeping mutation will take effect.

Apart from the fact that a simulation of too large numbers of individuals is not feasible, computationally expensive models make sensitivity analysis and model fitting more cumbersome. Two methods can overcome this [116]: (1) a simulation of one or several statistically representative volume elements of the larger system in full detail, whereas its size depends on the spatial variation of the features of interest; (2) a simulation of super-individuals that represent a group of similar individuals. In large populations, stochastic differential equation models may better describe heterogeneity. Besides limitations in modeling large-scale systems, IBMs can become too complex to analyse mathematically, understand and communicate. As otherwise too simple models might not be representative for natural systems, an intermediate model complexity is supposed to be optimal [123].

Good modeling practice is to use a structured and standardized description to present a model, e.g. to adopt the ‘ODD’ (overview, design concepts, and details) protocol for the description of IBMs [124], to check structural realism through the application of a pattern oriented modeling approach, which refers to a comparison of multiple simulated patterns with empirical patterns at varying scales, i.e. involving individual- and population-level characteristics [123], [116]. The structural realism of models should also be examined by model robustness tests. These involve parameter sensitivity analysis that identifies important parameters, which, if achievable, should therefore be carefully estimated with empirical methods. Other robustness tests focus on the model structure, referring to a sensitivity analysis in which processes are systematically included or omitted [125], [126]. Potential pitfalls of IBM are that individuals have global knowledge, processes that are important for the research question are ignored or something that should be an emergent property is imposed [116]. For example, a single donor cell should not be able to identify the last recipient in a population that is composed of thousands of completely mixed cells. Instead, a donor might only by chance select the ’right’ recipient cell for a transfer attempt. Another negative example could be that a study aims to investigate how bacterial interactions lead to spatial clusters, which in reality are induced by a predetermined, fixed spatial distribution of some nutrients.

### 4.5 Implementing conjugation – details that can make a difference

Leclerc et al. [111] reported that most of the studies they included within their review modelled horizontal gene transfer as a mass action process. According to this, the rates of change are often calculated according to the product of the transfer rate γ, the density of donors D (plasmid-bearing cells) and the density of recipients R (plasmid-free cells) such that transfer T=γRD, whereas T reflects a density estimate for the proportion of infected cells. Here it might become obvious that this way of modeling horizontal gene transfer is based on the assumption that the actual transfer rate is a constant. But in principle this is only the case if, apart from cell density, all factors that are known to influence the transfer rate are constant. In most cases, however, changes such as in nutrient availability can be expected, which means that the growth rate of the bacteria is not constant and this is known to have an influence on the conjugation rate [40], [127], [113], [93]. Therefore, it has been suggested to model the conjugation rate with a nonlinear substrate dependent expression similar to Monod kinetic or the Michaelis–Menton equation for enzyme kinetics [128]. If substrate availability is not explicitly considered in the model, a simple logistic factor f can be used instead [30], such as it is often used to model declining growth rates of bacteria approaching the maximal cell density N (carrying capacity)with f=1-(D+R)/N. Thus, a corrected estimate of the transfer rate $T_{\mathrm{corr}.}$ can be obtained simply by including f such that $T_{\mathrm{corr}.}=f\gamma \mathrm{RD}$. This principle can also be applied to stochastic, spatially explicit models in which the local availability of vacant sites can mimic the local resource availability that affects both bacterial growth and conjugation rates [101]. Such alignment of growth and conjugation rates with the factor *f* might be negligible under certain circumstances. However, if a population is simulated that is approaching carrying capacity in its growth, then models that do not couple the conjugation rate to growth predict that the bacteria can no longer grow, but the conjugation rates are at their maximum.

## 5 Summary and outlook

The conditions and processes affecting the persistence and occurrence of plasmid-coded antibiotic resistance are manifold, including many ecological characteristics and properties inherent to the plasmid types themselves. This study provided an overview and ecological framework for the study of plasmid dynamics with simulation models.

If the dissemination of antibiotic resistance by plasmids is addressed, the general purpose of a model is to describe the two dimensions of plasmid fitness, i.e. vertical and horizontal transmission. This can be done considering different degrees of complexity with respect to its abiotic and biotic dimension.

Model-based simplification is both a necessity and a valuable insight into key system features. However, a simplification only becomes visible when it is compared with the actual complexity of the system. Assumptions of models should therefore be clearly addressed and justified. In addition to uncertainty and robustness analyses, documentation and description of the step-by-step model development have great potential to improve the understanding of the properties and the behavior of a system, because this learning process provides important insights into the rationale of the decisions made for simplification and their alternatives, which are often not explained in detail.

Biotic interactions, evolutionary modificationsas well as individual heterogeneity and dynamic adaptations can have a strong influence on the spread of plasmid-coded antibiotic resistance. The great advantage of individual-based models is that they describe the processes at the level where they actually take place: the individual cell. Therefore, these processes can be formulated much easier and can be understood more easily by non-modelers. The combination of such computer-based tools with the increasing number of laboratory methods that provide detailed insights into the dynamics of individual cells offers a way to address the manifold open research questions that are related to the complexity of natural environments.

Models can serve as a powerful tool to understand the environmental dimension of the spread of antibiotic resistance and help to design ’ecology-and-evolution-proof’ counteractive strategies.

## Declaration of Competing Interest

The author declares to have no known competing financial interests or personal relationships that could have appeared to influence the work reported in this paper.
